# Three Drinking-Water–Associated Cryptosporidiosis Outbreaks, Northern Ireland

**DOI:** 10.3201/eid0806.010368

**Published:** 2002-06

**Authors:** Scott Glaberman, John E. Moore, Colm J. Lowery, Rachel M. Chalmers, Irshad Sulaiman, Kristin Elwin, Paul J. Rooney, Beverley C. Millar, James S.G. Dooley, Altaf A. Lal, Lihua Xiao

**Affiliations:** * Centers for Disease Control and Prevention, Atlanta, Georgia, USA; † Belfast City Hospital, Northern Ireland, United Kingdom; ‡ University of Ulster, Coleraine, Northern Ireland, United Kingdom; § Singleton Hospital, Swansea, Wales, United Kingdom

**Keywords:** *Cryptosporidium parvum*, genotyping, subgenotyping, outbreak, epidemiology, Northern Ireland

## Abstract

Three recent drinking-water–associated cryptosporidiosis outbreaks in Northern Ireland were investigated by using genotyping and subgenotyping tools. One *Cryptosporidium parvum* outbreak was caused by the bovine genotype, and two were caused by the human genotype*.* Subgenotyping analyses indicate that two predominant subgenotypes were associated with these outbreaks and had been circulating in the community.

Human cryptosporidiosis is predominantly caused by the human and bovine *Cryptosporidium parvum* genotypes, which differ in host range; the former infects mostly humans under natural conditions, and the latter infects both humans and some farm animals such as cattle, sheep, and goats (*1*). In many geographic areas, both *C. parvum* transmission cycles can occur in humans, but the importance of each genotype as a source of human infection probably varies (*2*–*4*). Both genotypes have been involved in waterborne outbreaks of human cryptosporidiosis in the United States, Canada, and the United Kingdom (*2*,*5*,*6*).

From April 2000 to April 2001, three drinking-water–associated outbreaks of cryptosporidiosis occurred in Northern Ireland. These outbreaks were epidemiologically unrelated and originated from geographically separate areas. Concerns have been raised about a possible relationship between *C. parvum* genotypes and subgenotypes associated with these outbreaks. In this study, for genotyping analysis, we investigated these outbreaks using a small subunit rRNA (SSU rRNA)-based polymerase chain reaction-restriction fragment length polymorphism (PCR-RFLP) genotyping tool, as well as the *Cryptosporidium* oocyst wall protein (COWP) PCR assay. For subgenotyping analysis, sequence typing of the 60-kDa glycoprotein (GP60) was used.

## The Study

The three drinking-water–associated outbreaks occurred in the greater Belfast area. Outbreak A occurred during April and May 2000; at least 129 cases were laboratory confirmed. Outbreak B occurred in August 2000, involving at least 117 cases. Outbreak C occurred in April 2001; at least 230 people were infected (*7**–**9*; unpub. data). An outbreak patient was defined as a person with microscopically confirmed *Cryptosporidium* infection who became ill during the outbreak period and who was a resident in the water supply areas. The attack rates for outbreaks A, B, and C were 34, 180, and 58 cases/100,000 persons, respectively. Outbreak B was thought to be caused by the ingress of human sewage from a septic tank into the drinking water-distribution system and C from the ingress of wastewater from a blocked drain.

For molecular analysis, 34, 42, and 44 microscopically positive stool samples from outbreaks A, B, and C, respectively, were used. One wastewater sample from a blocked drain implicated in outbreak C was also analyzed. Control isolates of the *C. parvum* genotypes were also included in the subgenotyping analysis. Fourteen control isolates were from sporadic *C. parvum* infections of the bovine genotype in a rural area in west Ireland about 100 miles from Belfast, and the water supply was entirely different. Ten control isolates were from sporadic *C. parvum* infections of the human genotype in northwest England during the same time as outbreak C.

*C. parvum* genotype in human fecal samples was first determined by a COWP gene-based PCR-RFLP tool (*10*). Oocyst suspensions were prepared from feces by using salt flotation (*11*). The oocysts were washed and resuspended in deionized water and stored at 4°C before use. To extract DNA, oocyst suspensions were incubated at 100°C for 60 minutes, digested with proteinase K (3 mg/mL) in lysis buffer at 56°C for 30 minutes, and extracted by spin-column filtration (QiAMP DNA kit, Qiagen, Crawley, UK). Extracted DNA was stored at -20°C before use. Genotypes were investigated by using the COWP gene primers cry15 and cry9 to amplify a 553-bp region, which was then subjected to endonuclease digestion by *Rsa*I (*10*).

Genotypes were confirmed by using an SSU rRNA-based PCR-RFLP tool (*12*). Subgenotyping was done by sequence analysis of the GP60 gene (*13*). Before molecular analysis, the wastewater sample was processed by both salt flotation (*11*) and immunomagnetic separation (Dynal, Lake Success, NY), following the manufacturer-recommended procedures (*14*). Both genotyping and subgenotyping tools used nested PCR amplification of targeted genes. The primers used for GP60 were 5´-ATA GTC TCC GCT GTA TTC-3´ and 5´-TCC GCT GTA TTC TCA GCC-3´ for primary PCR and 5´-GGA AGG AAC GAT GTA TCT-3´ and 5´-GCA GAG GAA CCA GCA TC-3´ for secondary PCR. The PCR reaction contained 1X Perkin-Elmer (Norwalk, CN) PCR buffer, 3 mM MgCl_2_, 200 µM (each) deoxynucleoside triphosphate, 200 nM of the forward and reverse primers, 5 units of *Taq* polymerase, and 0.5–2 µL of DNA template (for primary PCR) or 2 µL of primary PCR product (for secondary PCR) in a total 100-µL reaction mixture. Each PCR reaction was then subjected to 35 cycles of denaturation at 94°C for 45 seconds, annealing at 50°C for 45 seconds, and extension at 72°C for 60 seconds, with an initial denaturation at 95°C for 3 minutes and a final extension at 72°C for 10 minutes. PCR products were sequenced in both directions on an ABI3100 (Applied Biosystems, Foster City, CA) with forward and reverse primers. An additional sequencing primer (5´-GAG ATA TAT CTT GGT GCG-3´) was used in the sequencing of GP60 PCR products. We aligned the study’s GP60 nucleotide sequences with each other and with sequences from the GenBank database with GCG software (Genetics Computing Group, Madison, WI). A neighbor-joining tree was constructed from the aligned sequences as described (*15*).

Thirty-three of the 34 stool samples from outbreak A were amplified by both the COWP and SSU rRNA-based nested PCRs. RFLP analysis of the PCR products showed that all 33 PCR-positive samples had the *C. parvum* bovine genotype. Thirty-two of the 42 stool samples from outbreak B were also positive by PCR, and all belonged to the *C. parvum* human genotype. Furthermore, in outbreak C, 36 of 44 samples had the *C. parvum* human genotype, and 8 had the bovine genotype. After further epidemiologic investigations, these eight bovine genotypes, although submitted to the primary diagnostic laboratory at the same time as the human genotypes, were considered contemporary sporadic cases and not part of outbreak C. These patients did not live in the distribution area of the water supply implicated in the outbreak. The patients lived in southern Down County, whereas the outbreaks occurred in southern Antrim County and northern Down County. Results of the two genotyping methods were in complete agreement in both detection rates and genotyping result.

Subgenotype analyses of the GP60 gene showed that of the 30 stool isolates of the *C. parvum* bovine genotype examined for outbreak A, 25 isolates belonged to a single GP60 subgenotype and 5 isolates belonged to another subgenotype. In contrast, 14 samples of the *C. parvum* bovine genotype isolated from sporadic cases of human cryptosporidiosis from west Ireland, which were unrelated to any of the Northern Ireland outbreaks, belonged to nine subgenotypes. Subgenotype analysis of 31 stool samples from outbreak B showed the presence of only one subgenotype of the *C. parvum* human genotype. For outbreak C, all 36 *C. parvum* human genotype stool isolates were identical to the subgenotype involved in outbreak B. In addition, all eight *C. parvum* bovine genotype stool isolates, which were contemporary with, but not from, the area affected by the outbreak, were identical to the predominant subgenotype in outbreak A. The wastewater sample from the blocked drain implicated as the cause of outbreak C contained oocysts of the same subgenotype as the *C. parvum* human genotype. Of the nine sporadic isolates of the *C. parvum* human genotype from northwest England, eight belonged to the same subgenotype as the *C. parvum* human genotype involved in outbreaks B and C (Figure). Most infected persons each had only one genotype/subgenotype of *C. parvum*, judged by the RFLP profile, the absence of underlying signal in the chromatogram of the sequencing result, and at least five independent PCR analyses of each sample. The SSU rRNA technique can detect multiple *Cryptosporidium* parasites in individual samples (*16*).

**Figure:**
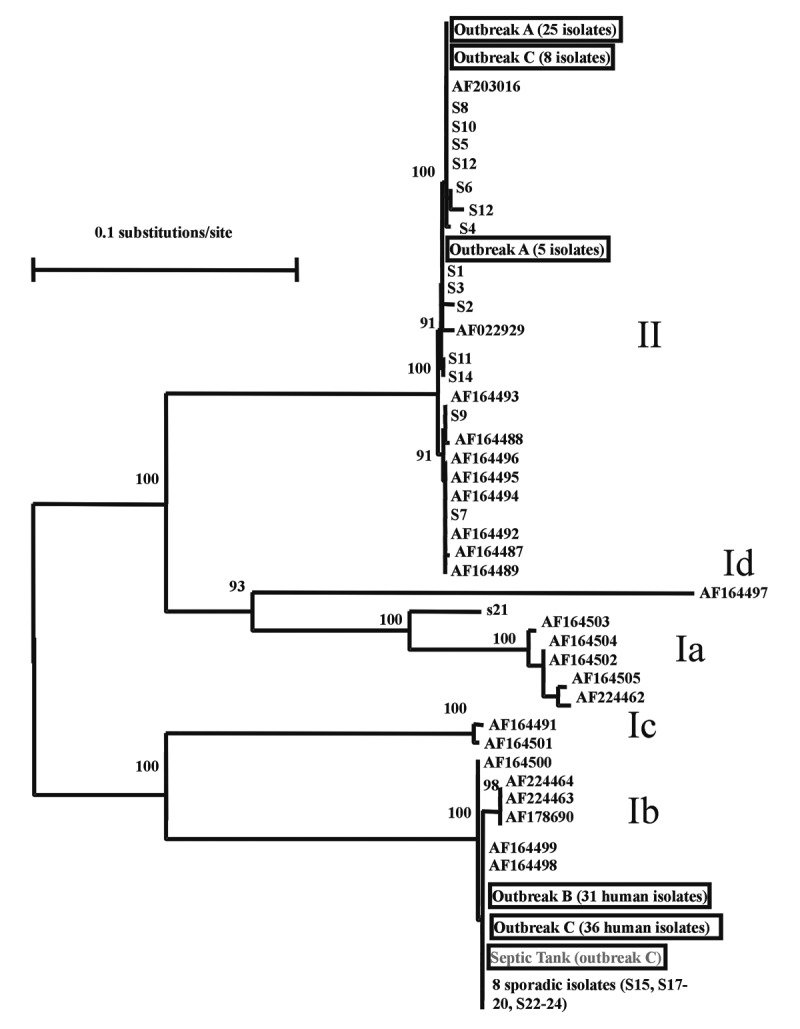
Genetic relationship among *Cryptosporidium* parasites found in three Northern Ireland outbreaks (outbreaks A, B, and C), sporadic cases in west Ireland (S1 to S14) and the northwest of England (S15 to S24), subgenotypes described by Strong et al. (*11*), and an unpublished sequence (AF203016) from the GenBank database. The isolates with accession numbers were mostly human and bovine and from the United States with the exception of AF164488, AF164492, and AF164493, which were isolated from humans in Zaire, Peru, and Brazil, respectively, but had been passaged in calves in the United States. Nomenclature for groups of subgenotypes is adapted from Strong et al.: Ia, Ib, Ic, and Id for subgenotypes of the *C. parvum* human genotype and II for subgenotypes of the *C. parvum* bovine genotype (*11*). Data presented are a neighbor-joining tree of GP60 sequences.

## Discussion

Results of genotyping analysis support epidemiologic observations that these three drinking-water–associated outbreaks of cryptosporidiosis in Northern Ireland were unrelated, although they all occurred in the greater Belfast area over a 1-year period. Outbreak A was caused by the *C. parvum* bovine genotype, and outbreaks B and C were caused by the *C. parvum* human genotype. The occurrence of the *C. parvum* human genotype in outbreaks B and C suggests that these two outbreaks were, at least in part, caused by contamination of the drinking-water supply by seepage of raw sewage and through wastewater into the drinking water distribution systems, respectively. This finding illustrates the value of timely genotyping analysis during outbreak investigations. The source of contamination is further supported by subgenotyping analysis of the wastewater sample from the blocked drain that was epidemiologically implicated in outbreak C. This sample contained one subgenotype of the *C. parvum* human genotype indistinguishable from the subgenotype found in most infected persons.

The failure to detect *Cryptosporidium* in 10 of the microscopically positive samples in outbreak B was most likely not because of rare *Cryptosporidium* genotypes; the SSU rRNA technique is *Cryptosporidium* genus specific and detects all known *Cryptosporidium* spp. (*12*,*14*–*16*). The presence of PCR inhibitors in the extracted DNA may have prevented the detection of *Cryptosporidium* by PCR.

Results of subgenotyping analysis nevertheless indicate that the three recent cryptosporidiosis outbreaks in Northern Ireland were caused by two predominant subgenotypes of *C. parvum* that probably had been circulating in the community before the outbreaks. These two subgenotypes of *C. parvum* are also the most common subgenotypes found in Northern Ireland and northwest England. The human subgenotype was found in 8 of 9 sporadic isolates from northwest England and the bovine subgenotype in 4 of 14 isolates in another part of Ireland.

The two subgenotypes of the *C. parvum* bovine genotype found in outbreak A and concurrent with outbreak C have not been found in most other areas (*3*,*4*). The only *C. parvum* isolate identical to one of the subgenotypes is an unpublished sequence (AF2030016) deposited in GenBank (Figure). The source of the other genotype, however, is unknown. In contrast, the subgenotype of the *C. parvum* human genotype involved in outbreaks B and C has a wide geographic distribution, with isolates from United States, Canada, United Kingdom, Portugal, and Peru (*3*,*4*). This subgenotype, the most common subgenotype of the *C. parvum* human genotype found in the United States, was responsible for several waterborne and foodborne outbreaks of human cryptosporidiosis (*3*). This subgenotype has a worldwide distribution and is the cause of many outbreaks. Whether the wide distribution of this subgenotype of the *C. parvum* human genotype and apparent association with multiple outbreaks in geographically distinct areas result from unusual biologic fitness of this parasite is unknown.
